# “Mesmerizing and Terrifying”: Senusret III’s Unique Macrotia

**DOI:** 10.7759/cureus.51467

**Published:** 2024-01-01

**Authors:** Matthew D Turner, Hänel J Eberly

**Affiliations:** 1 Emergency Medicine, Penn State Health Milton S. Hershey Medical Center, Hershey, USA; 2 Medical School, Penn State College of Medicine, Hershey, USA

**Keywords:** external ear deformity, ancient egypt, senusret iii, medical history, macrotia

## Abstract

The ancient Egyptian pharaoh Senusret III was a legend to both his contemporaries and his descendants: an ideal of kingly power whose legacy of control and intimidation was remembered for centuries. Of particular note is the unique macrotia that the king's statues display. In this paper, we discuss possible etiologies of Senusret's unique presentation and ultimately conclude that Senusret's immortalized features were likely rooted in propaganda rather than a medical cause.

## Introduction and background

In his writings on the history of Egypt, the ancient Greek historian Herodotus wrote of a legendary pharaoh who had lived centuries earlier. Well over a millennium after his passing, the ruler that Herodotus described as “Sesostris” was remembered by the Egyptians as an unmatched ideal of kingly power [1], a great conqueror who had spread his domain to an unprecedented scale through an unstoppable campaign of expansionist imperialism [2]. Some legends depicted him as a conqueror who had crushed all of Asia, even greater than Alexander the Great [1]. Although it often exaggerated his exploits into hyperbole, the myth-making of later generations had a strong basis in the truth. Senusret III, also known as Senwosret III, was one of the most powerful pharaohs of ancient Egypt’s Twelfth Dynasty. In a lineage that saw a massive centralization of authority and the expansion of the state’s power, Senusret III was notable among his peers for the heights of his ambition and the ruthlessness with which he pursued his goals [3].

## Review

Senusret III

Born to Senusret II and his sister-wife, Khenmetneferhedjet-waret [4], Senusret III rose to the Egyptian throne in approximately 1836 BCE [3]. He quickly perfected the “despotic model of monarchy” that his predecessors of the Twelfth Dynasty had developed [3], and almost immediately set his scribes and poets on a massive propaganda campaign that saw some of ancient Egypt’s most famous works of literature. Pieces such as The Cycle of Hymns, The Complaints of Khakheperraseneb, and The Admonitions of Ipuwer, all compositions that featured significant propaganda praising the monarchy’s endless virtues, were specifically targeted at the literate upper class. Simultaneously, Senusret tightened his grip on the powerful provincial governors who ruled over the banks of the Nile. In the past, these governors, also known as nomarchs, had torn the kingdom apart when a series of weak kings had allowed them to gather too much power. Ceding over power was not a mistake that Senusret would ever make. Throughout his reign, he successfully beat and bullied both his immediate circle and the provincial elites into utter submission to the throne [3]. In a single generation, the previous model that had once ruled Egypt for centuries, nomarchs controlling individual provinces, while paying lip service to a weak and ineffective pharaoh, had completely vanished [3]. Even the private tombs that the nomarchs had once constructed in their provinces vanished, as the once-powerful elites frantically jostled one another to be buried as close to the royal court as possible [5]. Only Senusret’s hands could be allowed to steer the ship of state [3].

While literature and political maneuvering served to keep the kingdom’s elites in line, control over the kingdom’s numerous and widely illiterate population required different strategies. Like his forebears, Senusret set out on a series of ambitious building projects that showcased the “obsession with rigid planning” that characterized much of the Twelfth Dynasty [3]. He soon had a pyramid constructed at Abdju, and to house his workers, he had a mathematically laid out town built near it called Wah-sut-Khakaura-maa-kheru-em-Abdju, meaning “enduring are the places of Khakaura (Senusret’s throne name), the justified, in Abdju” [3]. The state enacted a program of surveillance, particularly on the southern borders, where “in an atmosphere of nervousness approaching paranoia”, patrols constantly roamed the countryside, stopping and searching locals at will [3]. The dispatches that his commanders sent to their monarch frequently ended with the phrase: “All the affairs of the King’s Domain (life, prosperity, health!) are safe and sound” [3].

But it was not enough to simply secure the South. In the 8th, 10th, 16th, and 19th years of his reign, Senusret launched a series of bloody campaigns into Nubia, expanding the Egyptian kingdom over an unprecedented amount of territory [5]. A series of mighty fortresses with names like “Destroying the Nubians”, “Subduing the foreign lands”, and “Suppressing the Nubians”, secured Egyptian control over its broken southern neighbor [3]. At the time, the forts were both military and logistical marvels, providing an integrated and unprecedented system to support Egyptian occupation [3]. The legacy of Senusret’s campaigns would last for centuries; eventually, he would be venerated as a God in Wawat, the ever-shifting border between the rival kingdoms [3]. “His Majesty’s tongue restrains Nubia,” inscriptions praising the king boasted, “His utterances make the Asiatics flee” [2].

Although his accomplishments in propaganda, surveillance, conquest, and control already outpaced the other members of the Twelfth Dynasty, Senusret III also developed a new tool for projecting his power across Egypt: portrait sculpture. As Wilkinson notes, “Never before in the history of ancient Egypt had a king used sculpture so effectively to project so terrifying an image of royal power” [3]. Where previous pharaohs had official statues that portrayed them with eternally youthful, idealized portraits, Senusret was the first to adopt a new realistic sculptural style. This “frank portrayal” was shocking to his contemporaries, depicting “protruding ears, rounded, projecting eyes with prominent lids, pouches beneath the eyes… and a generally downturned mouth with mounds of flesh at the sides” [6]. His face also featured worry lines, and bulging, hooded, eyes. The king is often depicted with deep nasolabial folds, giving a distinctive “disconsolate look” [7]. While the king always retained a “taut, muscular, and virile” torso reminiscent of a young warrior in his prime, his face - radically and unnaturally realistic, by contemporary standards - was at once “mesmerizing and terrifying” to those who viewed it [3]. Adding to the effect, each of the statues had to be treated with the same awe and reverence that one might reserve for the pharaoh himself, as an “individual divinity worthy of its own offerings” [5]. Just as terrifying was the sheer quantity of statues made, many remain to this day [3], and in many temples, Senusret “tripled or quadrupled his presence, adding multiple statues, each with the same stature, yet none exactly the same....he seemed everywhere at once, his statues terrifying in their mastery of stone” [5]. The upper class of Egypt followed their pharaoh’s example, mimicking his unique features in their statutory. However, none of them dared to depict themselves with the same muscular body as Senusret III, for all power, symbolic and otherwise, belonged in the hands of no one but the pharaoh. The best that they could hope for was to be represented as extensions of the king’s body, “watching on his behalf” [5]. The purpose of such terrifying imagery was quite blatant. In one of his fortresses established in Nubia, Senusret established a statue of himself in a special shrine for his soldiers. The inscription reads: “My Majesty has had an image of My Majesty made upon this frontier… so that you will be steadfast for it, so that you will fight for it” [3]. Even on the utmost borders of his domain, the king’s intimidating visage elicited a powerful mix of reverence and fear [3].

**Figure 1:**
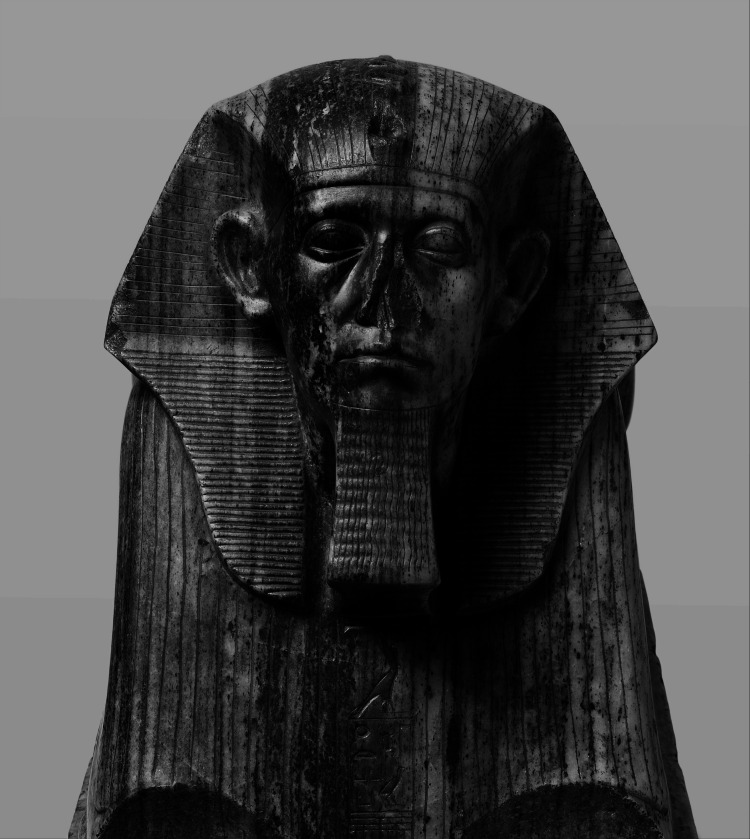
An image of Senusret III as a sphinx This work was obtained from the Metropolitan Museum of Art and is part of the Public Domain [10].

**Figure 2:**
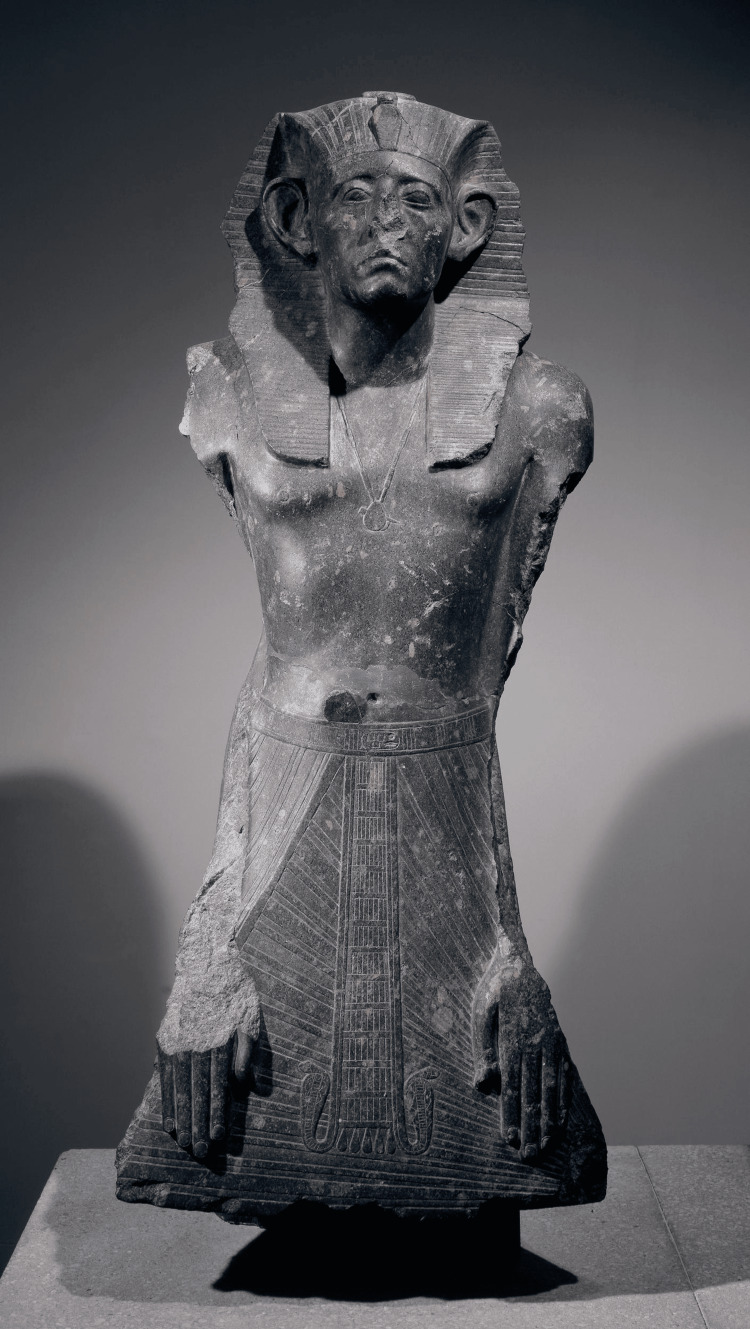
Granodiorite statue of Senusret III Obtained from the British Museum under the CC BY-NC-SA 4.0 license [11].

**Figure 3:**
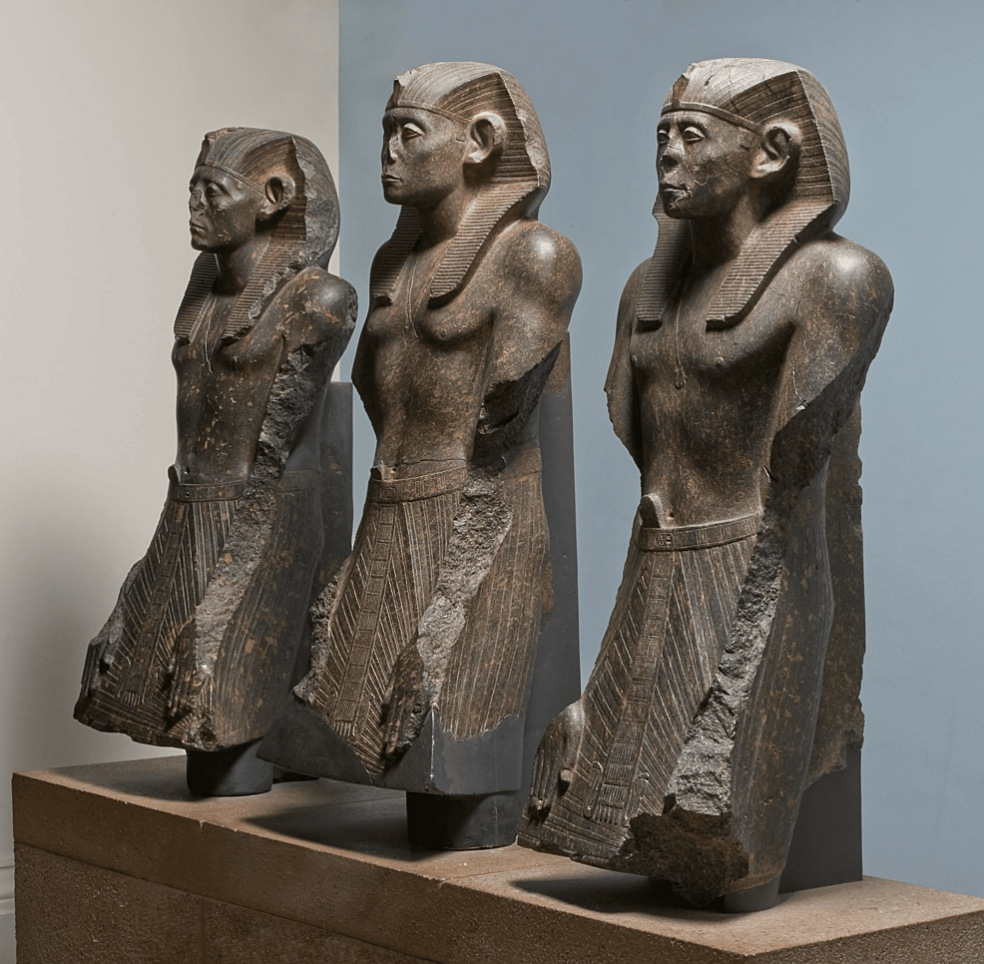
A series of statues featuring Senusret III Obtained from the British Museum under the CC BY-NC-SA 4.0 license [12].

Senusret’s features are particularly notable for one trait in particular - his enlarged ears, predominately displayed across a majority of his imagery [4], as shown in Figures 1-3. Senusret’s statutory evolved over his reign, from very youthful statues to ones that appear as a man in his sixth or seventh decade of life [8]. Even in the earliest years of his rule, the monarch was depicted with enlarged, high-set ears [4]. Given the new school of realistic sculpture that Senusret brought to the forefront of Egyptian society [3], as well as his desire, both cultural and religious, to preserve his individual identity for all time [9], it is possible that these depictions were consistent with the king’s actual features. Given this, we propose that there may be a medical explanation for Senusret’s unique appearance in the archaeological record.

Possible explanations

Macrotia

Congenital anomalies of the external ear are rare and exhibit a broad spectrum in terms of type and severity. Prominent ears, sometimes known as macrotia or protruding ears, are one of the most common deformities and may affect up to 5 percent of the population [13,14]. In a normal human ear, the superior aspect of the ear usually approximates the height of the brow, and the width of the ear correlates with approximately 50-60 percent of its length [14,15]. In macrotia, the upper third of the ear is most often elongated rather than the lobe [16,17]. Ear protrusion is related to the concho-scaphal angle (the angle of the ear in relation to the head), and can occur when the conchal bowl is overdeveloped, the antihelical fold is poorly developed, or a combination of these features [18].

Senusret’s imagery shares many of these characteristics. Statues of the king recovered at Deir el-Bahari show a distinct overgrowth of the upper concha [4]. Many other statues, particularly those produced in the kingdom’s productive Theban workshops, depict the king with an exaggerated angle of inclination between the ears and the cheeks [4], consistent with modern definitions of macrotia [18].

Syndromes

While prominent ears by themselves do not cause hearing impairment, approximately 30 percent of external ear deformities are associated with syndromes, including additional malformations [19]. Examples of syndromes with related ear deformities include Treacher-Collins syndrome, Crouzon syndrome, Apert syndrome, Wildervanck syndrome, and chromosomal abnormalities such as trisomy 13, trisomy 18, and trisomy 21, among others [19]. While syndromic ear malformations demonstrate autosomal recessive inheritance in 90% of cases, non-syndromic ear deformities such as microtia show a slightly different distribution, with most cases being attributed to autosomal dominant inheritance [19,20]. Various studies of inner ear development have demonstrated the role of various molecules, such as transcription factors, genes, growth factors, and cell adhesion proteins in ear malformations [19]. Neither Senusret III’s father, Senusret II, nor his mother, Khenmetneferhedjet-waret, seem to share his appearance of enlarged ears, which could suggest a spontaneous genetic mutation rather than an inherited or syndromic cause. However, this possibility also remains unlikely. The majority of syndromic ear malformations and genetic abnormalities are associated with microtia or acrotia rather than macrotia [19], and hearing changes are very rarely associated with macrotia or protruding ears.

While genetic factors may play a cause, a significant number of acquired ear malformations may arise from exogenous factors during pregnancy, including viral infections such as cytomegalovirus, herpes simplex virus, rubella, toxoplasmosis, and poliomyelitis [19]. Other factors are as varied as malnutrition, hypoxia, Vitamin A deficiency, alcohol, and even noise exposure [19]. In cases such as Pendred syndrome, thyroid hormone deficiency in a pregnant mother may lead to ear malformations of the fetus [19]. Many of these injuries occur during embryogenesis when the pinna of the ear, along with the tragus, crus helices, and upper helix, develop from the first branchial groove at approximately 40-45 days following conception. This process is completed approximately four months after conception [19]. It is possible that Senusret III may have been exposed to an exogenous exposure that affected the development of his outer ears during this period of development. Unfortunately, over 3000 years since the pharaoh’s reign [3], it is impossible to determine what teratogens he may have been exposed to. Despite this, teratogenic factors may be reasonably ruled out; approximately 70-90% of malformations of the outer and middle ear are unilateral [19], and the vast majority are present with either microtia or macrotia [19]. Given this, it is likely that Senusret’s depicted macrotia was due to another process entirely.

Trauma

Senusret III prided himself as a warrior-king and often bragged of his conquests. His monuments often featured declarations of his ferocity and ruthlessness - “I have carried off their women and brought away their dependents, burst forth to poison their wells, driven off their bulls, ripped up their barley, and set fire to it,” he proudly announced upon the conclusion of a campaign against Nubia [3]. Even for pharaohs, life in ancient Egypt was not always an easy one. It was not unknown for the highest rulers of Egyptian society to fight in the front lines; later figures such as Taa fought and died in combat, their mummies still bearing the blows and wounds that felled them in life [3]. Physical education was often stressed just as much as intellectual pursuits for the future rulers of Egypt, and elites were often expected to participate in sports such as wrestling, rowing, running, and swimming [3]. Given his proclivity for warfare and likely upbringing, it is possible that Senusret III may have experienced trauma to his ears as a young man.

A significant cause of ear deformities includes exogenous force and trauma; contemporary examples include bites (both animal and human), traffic accidents, and burn injuries [21]. Similar traumatic accidents would have been present in the violent world of ancient warfare. However, the bilateral nature of Senusret III’s macrotia detracts from this theory, as exogenous force would be more likely to cause asymmetrical defects [21]. Interestingly, a number of Senusret’s statues depict him wearing headgear that would have offered his ears a degree of protection during combat [4]. While members of the Egyptian royal family often participated in warfare at young ages [3], Senusret's bilateral macrotia suggests that this is unlikely.

Classic "cauliflower ear", as often experienced by boxers and wrestlers, is a rare exception, and may often present with deformities to the bilateral ears. However, these deformities, secondary to repeated trauma leading to scarring and improper regeneration of the auricular cartilage, are characterized by "thickening, irregular projection of the anterior ear and distortion of the auricular outline" [22]. Senusret's ears, while depicted as enlarged in his statutory, show no signs of visible deformity or distortion associated with this phenomenon [4].

Arterio-venous malformations

Although rare, macrotia has been identified in relation to congenital arterio-venous malformations (AVM) [23,24]. The most common sites of AVMs in the face include the cheek, ear, lips, nose, and forehead, and usually involve blushing of the skin or the presence of a birthmark [25]. While this is an interesting and possible cause of macrotia, it is unlikely to have affected Senusret III as his ears were bilaterally enlarged [4], and there are no reports of him being affected by any similar conditions.

Aging

The role of natural aging on the ear should be considered. Earlobes naturally elongate with age due to the natural breakdown of collagen and elastin in the skin. This can become even more obvious with the use of earrings and other ear accessories, jewelry Senusret III and other pharaohs of the time would have used often [26].

However, this theory can also be discounted. While the rest of his features display a spectrum that appears to subtly change over the progression of his reign, possibly consistent with the king’s actual physical aging [8], his ears appear to be consistently enlarged [3].

Propaganda

Of all the theories that we propose, propaganda is the most likely. From the earliest days of his reign, Senusret III displayed a remarkable talent for mythologizing [3] and skillfully developed a cult of personality that lasted for centuries [2]. Given this, the depiction of his enlarged ears was likely symbolic, a message that the king was all-hearing, a warning consistent with the powerful police state apparatus that he strengthened and enlarged [3]. While this depiction was a new innovation for Egyptian rulers, it had already been down centuries earlier by rulers such as Gudea of Lagash in nearby Mesopotomia. Notably, Gudea’s depiction of enlarged ears was “meant to show him as a wise and attentive leader” [4], a depiction with less intimidating connotations than Senusret’s visage.

It is likely that Senusret’s imagery did have some basis in reality - there does appear to be a correspondence between his features and the king’s aging throughout his reign [8]. However, only a tiny fraction of the Egyptian population would have ever seen Senusret in person. Given this, it would have been in the king’s interest to alter his features as needed for propaganda purposes [4]. Even the unique haggard appearance of the pharaoh could have served as a metaphor for ‘intellectual strength in maturity”, in a culture that associated age with knowledge, while his slightly bulging eyes symbolized eternal vigilance [6]. Senusret’s actual features remain unknown to the present day, as his mummy has never been found [4].

## Conclusions

The realistic depictions of Senusret III, unprecedented in the records of ancient Egypt, are notable for his depiction of macrotia. While a number of medical etiologies are possible, including trauma, congenital syndromes, arterio-venous malformations, and aging, we ultimately conclude that Senusret’s macrotia is a product of propaganda designed to reinforce the image of the pharaoh as an all-hearing monarch.
